# Cardio-oncology: need for novel structures

**DOI:** 10.1186/s40001-018-0359-0

**Published:** 2019-01-03

**Authors:** Lars Michel, Tienush Rassaf

**Affiliations:** Department of Cardiology and Vascular Medicine, West German Heart and Vascular Center, Medical Faculty, University Hospital Essen, Hufelandstr. 55, 45147 Essen, Germany

**Keywords:** Anthracyclines, Cancer, Cardio-oncology, Cardiotoxicity

## Abstract

Cancer and cardiovascular diseases are the main causes for morbidity and mortality in modern society. In the United States of America (USA), over 1.7 million new cancer cases will presumably be observed in 2018. Progress in cancer treatment has greatly improved survival and it is estimated that 15.5 million cancer survivors currently live in the USA. The number of cancer survivors is expected to increase by 68% until 2040. Moreover, the portion of cancer survivors at the age of 65 years or older will increase from 62% to approximately 73% in 2040 which in turn enhances comorbidities in cancer survivors. Increased survival and age of cancer patients has unmasked the burden of cancer and cancer therapy-associated cardiovascular diseases. Depending on cancer treatment modalities, early cardiovascular toxicity is observed in up to 48% of patients. Late cardiotoxicity can be found in 30% of patients at 13 years after cancer treatment. Cardio-oncology aims to identify cancer therapy-related cardiovascular side effects and to provide optimum multidisciplinary care for cancer patients. So far, scientific effort has generated a profound knowledge on underlying pathomechanisms and clinical implications but standardized recommendations and structural requirements for cardio-oncology care are still limited.

Cancer and cardiovascular diseases are the main causes for morbidity and mortality in modern society [1]. In the United States of America (USA), over 1.7 million new cancer cases will presumably be observed in 2018 [2]. Progress in cancer treatment has greatly improved survival and it is estimated that 15.5 million cancer survivors currently live in the USA. The number of cancer survivors is expected to increase by 68% until 2040 [3]. Moreover, the portion of cancer survivors at the age of 65 years or older will increase from 62% to approximately 73% in 2040 which in turn enhances comorbidities in cancer survivors [3]. Increased survival and age of cancer patients has unmasked the burden of cancer and cancer therapy-associated cardiovascular diseases [4]. Depending on cancer treatment modalities, early cardiovascular toxicity is observed in up to 48% of patients. Late cardiotoxicity can be found in 30% of patients at 13 years after cancer treatment [4, 5]. Cardio-oncology aims to identify cancer therapy-related cardiovascular side effects and to provide optimum multidisciplinary care for cancer patients [4, 6]. So far, scientific effort has generated a profound knowledge on underlying pathomechanisms and clinical implications but standardized recommendations and structural requirements for cardio-oncology care are still limited.

Anthracyclines have been identified as the prototype for cardiotoxicity in cancer therapy. This group of substances (e.g. doxorubicin, daunorubicin, epirubicin) is used in a variety of malignancies, e.g. breast cancer and hematologic cancers. Anthracyclines induce left ventricular (LV) dysfunction in 18–48% of patients [7]. The generation of reactive oxygen species and inhibition of topoisomerase II has been identified as the underlying pathomechanism of anthracycline-induced cardiotoxicity [5]. Several groups of patients receiving cancer therapy are at increased risk for development of cancer therapy-related cardiotoxicity. Particularly, cardiovascular risk of survivors of childhood cancer is highly elevated [8]. In pediatric patients treated with anthracyclines and radiotherapy, the overall risk for cardiac disease was 62-fold increased [8]. The median onset of cardiac disease was at the age of 29 years with a cumulative incidence of first diagnosis of cardiac disease of 11% at the age of 40 [8]. Despite these alarming data considering the young age of this group of patients, insufficient scientific effort has been undertaken so far to investigate potential cardioprotective measures and to implement standardized long-term cardio-oncologic monitoring of this collective into clinical routine [8]. Elderly patients with cardiovascular comorbidities represent a second group that is highly relevant for cardiotoxic side effects that is continuously growing with increasing age of cancer survivors [3, 4]. Here, presence of arterial hypertension, diabetes mellitus, smoking, anthracyclines and manifest coronary artery disease augment the risk for cardiotoxicity [4, 5, 9]. Lipid monitoring and management of modifiable risk factors is beneficial in the prevention of cancer therapy-induced cardiotoxicity [9]. However, a standardized approach (e.g. target serum lipid values) has not yet been established for cancer patients.

Within the last decade, targeted tumor therapy has partly replaced or complemented conventional chemotherapy. While at first no effect on the cardiovascular system was suspected, large-scale application of several targeted substances has revealed various significant cardiovascular adverse effects [10]. Anti-vascular endothelial growth factor receptor therapy increases the risk of cardiac ischemia and hypertension [11, 12]. Tyrosine kinase inhibitors have been successfully implemented in the treatment of metastatic melanoma but were shown to induce hypertension and LV dysfunction [13]. Patients treated with immune checkpoint inhibitors (ICI) are prone to autoimmune-triggered adverse events such as fulminant lymphocytic myocarditis in 1.14% [7, 14]. Considering these severe side effects of various new therapies that are increasingly implemented in cancer therapy, cardiotoxicity of novel therapeutics will be of particular importance for the management of future cancer patients.

Advancement in cancer treatment poses new challenges in cardio-oncology. Increased survival of cancer patients and the implementation of novel targeted therapeutics with specific cardiovascular toxicities will further increase the burden of cancer therapy-associated cardiovascular disease in the future. Multidisciplinary management of patients at risk for cancer therapy-related cardiovascular adverse events or manifest cardiotoxicity is critical for optimum patient care. Standardized diagnostic and therapeutic pathways for the management of these patients are urgently needed. The establishment of cardio-oncology guidelines is inevitable to ensure high-quality medical care. In this context, parameters for cardio-oncology consultation can be defined to improve interdisciplinary cooperation. In addition, target values and parameters for the monitoring of cardiotoxicity can be determined and procedures in the event of manifest cardiotoxicity should be established. To facilitate cardio-oncology service, structural requirements are necessary. Basic dedicated cardio-oncology services need to be available for all patients undergoing cancer therapy. An advanced cardio-oncology team with expertise for modern tumor therapeutics, e.g. ICI therapy or tyrosine kinase inhibitor therapy, is recommended for university hospitals and specialized oncology centers [6]. To implement comprehensive cardio-oncology care for smaller hospitals or resident doctors, cardio-oncology networks with dedicated consultant cardiologists and electronic cardio-oncology consultation services are recommended [6]. Close interaction between the treating oncologist and a cardiologist with additional expertise in cancer therapy-related toxicities is inevitable for the best possible care [6, 15]. Inclusion of patients into studies is essential aiming to promote scientific progress in the young field of cardio-oncology (Fig. 1) [6].

**Fig. 1:**
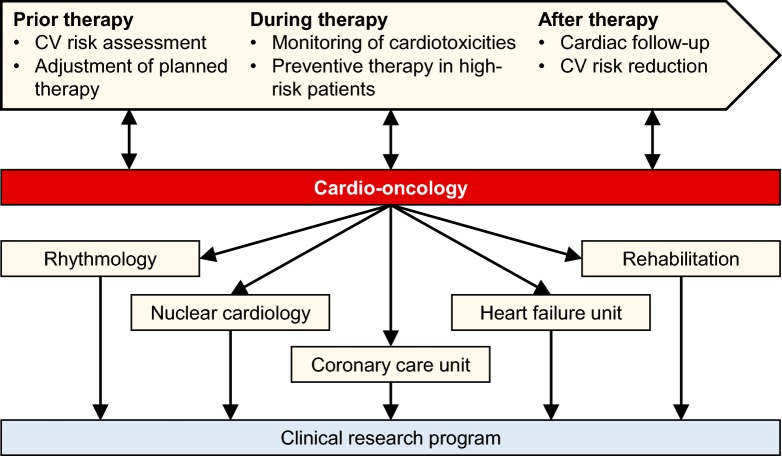
Workflow of a cardio-oncology unit. Treating oncologists identify patients with high risk for cardiovascular adverse events for cardio-oncologic consultation. The cardio-oncology unit conducts a baseline assessment before therapy, monitoring of cardiovascular toxicities during therapy and follow-up after therapy. Cardio-oncology benefits from multidisciplinary cooperation. Integration within a clinical research program facilitates scientific progress

At our institution, a cardio-oncology unit has been established as an in-hospital service and as an outpatient clinic. For the assessment of various forms of cardiotoxic effects, diagnostic pathways were created aiming to provide a standardized treatment of patients receiving conventional chemotherapy or different forms of targeted therapies. Baseline diagnostics including cardiac biomarkers and echocardiography with strain analysis, magnetic resonance imaging (MRI) and positron emission tomography–MRI are available for an individualized diagnostic assessment. The establishment of efficient cooperations and workflows within the involved departments appeared to be the most critical factor for the best possible cardio-oncology service.

The increasing demand for cardio-oncology care has led to the establishment of working groups within the European and German Society of Cardiology. However, a standardized advanced training and accreditation system for cardiologists working in the field of cardio-oncology is necessary to maintain high quality of cardio-oncology care [6]. Taken together, the implementation of cardio-oncology has greatly improved treatment of cardiovascular adverse events in cancer patients. Standardization of monitoring and treatment processes is now urgently needed to ensure optimum treatment quality.

## Abbreviations

ICI: immune checkpoint inhibitor
LV: left ventricular
MRI: magnetic resonance imaging
